# Comparison of the effects of metformin and empagliflozin on cardiac function in heart failure with preserved ejection fraction mice

**DOI:** 10.3389/fcvm.2025.1533820

**Published:** 2025-04-29

**Authors:** Xiehong Liu, Huiqi Zhao, Sisi Liu, Siao Wen, Wenjuan Fan, Qiong Xie, Bo Cui, Lin Zhou, Jianqiang Peng, Hongwei Pan, Zhaofen Zheng, Qinghai Zhang

**Affiliations:** ^1^ Department of Cardiology, Hunan Provincial People’s Hospital (The First Affiliated Hospital of Hunan Normal University), Changsha, Hunan, China; ^2^ Clinical Medicine Research Center of Heart Failure of Hunan Province, Changsha, Hunan, China; ^3^ Hunan Provincial Key Laboratory of Emergency and Critical Care Metabonomics, Institute of Emergency Medicine, Hunan Provincial People’s Hospital (The First Affiliated Hospital of Hunan Normal University), Changsha, Hunan, China; ^4^ Department of Emergency, Hunan Provincial People’s Hospital (The First Affiliated Hospital of Hunan Normal University), Changsha, Hunan, China; ^5^ College of Clinical Laboratory, Changsha Medical University, Changsha, Hunan, China

**Keywords:** heart failure with preserved ejection fraction, metformin, empagliflozin, cardiac fibrosis, cardiac hypertrophy

## Abstract

**Purpose:**

Recent evidence suggests that empagliflozin (EMPA) and metformin (MET) may improve prognosis in heart failure with preserved ejection fraction (HFpEF) patients. This study aims to compare their effects on cardiac structure and function in HFpEF.

**Methods:**

Male C57BL/6J mice were fed a high-fat diet with L-NAME for 8 weeks to induce HFpEF, followed by 4 weeks of MET or EMPA treatment. Cardiac structure and function were assessed. Network pharmacology and bioinformatics identified key targets, validated by RT-qPCR and WB.

**Results:**

EMPA-treated mice lost weight, unlike MET-treated ones. MET reduced systolic blood pressure significantly. Both treatments improved glucose tolerance; MET enhanced insulin sensitivity. EMPA increased exercise tolerance by extending exhaustion distance. Both treatments improved diastolic function, reduced heart weight, and attenuated myocardial fibrosis and hypertrophy. Plasma NT-proBNP levels were slightly elevated but not significant. EMPA downregulated HSP90 mRNA and protein expression; both drugs downregulated TGFβ.

**Conclusion:**

MET and EMPA improve cardiac fibrosis, diastolic function, and pulmonary congestion in HFpEF mice. MET acts by downregulating TGFβ, while EMPA affects collagen metabolism and downregulates HSP90 and TGFβ. These findings offer insights into HFpEF treatment.

## 1 Introduction

Heart failure with preserved ejection fraction (HFpEF) accounts for approximately half of all patients with heart failure (HF), and the prognosis of HFpEF is poor, with a 5-year survival rate as low as 35% (1). Epidemiological data revealed that the prevalence of HFpEF relative to heart failure with reduced ejection fraction (HFrEF) is increasing at a rate of 1% per year (2). While HFrEF has been studied extensively, much less is known about HFpEF, the ever-growing and predominant form of HF (3). Despite its increasing prevalence and clinical significance, HFpEF remains underexplored, particularly in terms of its therapeutic management and pathophysiology. Furthermore, A number of clinical trials have shown that traditional treatment drugs including angiotensin converting enzyme inhibitor/angiotensin II receptor blocker (ACEI/ARB), β-blockers, mineralocorticoid receptor antagonists and digoxin, failed to improve the prognosis of HFpEF (4–6).

Alterations in myocardial energy metabolism have been thought to contribute to the development of heart failure, and this is considered a potential therapeutic target for HFpEF, which remains inadequately addressed in existing treatments (7). Studies have found that reducing the uptake and oxidation of fatty acids, improving glucose oxidation, increasing ketone oxidation and branched-amino-acid metabolism can improve myocardial energy metabolism (8). At present, some antihyperglycemic drugs have been shown to reduce the rate of hospitalization and mortality in heart failure (9). Sodium-glucose cotransporter 2 (SGLT2) inhibitors are a new class of oral antidiabetic drugs that have been shown to reduce the risk of worsening heart failure or cardiovascular death in patients with HFrEF (10, 11). It has been recommended as the first-line drug for the treatment of HFrEF by several international heart failure guidelines (3, 12). The EMPEROR-Preserved study is the first to demonstrate a significant reduction in risk of cardiovascular death or hospitalization for heart failure (HHF) in HFpEF patients treated with SGLT2 inhibitor empagliflozin (EMPA) (13). Recently, a large number of studies and clinical trials have provided increasing evidence that EMPA can reduce the composite risk of cardiovascular mortality or hospitalization in HFpEF patients, regardless of the presence of diabetes (11, 13–15), suggesting that EMPA's beneficial effects may extend beyond its role in glucose metabolism to involve other molecular pathways relevant to HFpEF pathology.

Metformin (MET) is a widely prescribed antidiabetic drug with an established efficacy in the prevention and treatment of cardiovascular diseases. Its utility extends to patients with a range of cardiovascular conditions, including heart failure, pulmonary hypertension, myocarditis, and tumor cardiotoxicity, where it has been shown to effectively retard disease progression (16). A systematic review of 9 randomized controlled trials (RCTS) involving 2,486 patients with heart failure showed that MET did not affect left ventricular function and could improve myocardial oxygen consumption. Another randomized controlled trial also suggested that MET could improve cardiac contractility in non-DM patients with heart failure by reducing myocardial oxygen consumption (17). The retrospective cohort study conducted by Gu et al. demonstrated that prolonged exposure to metformin was linked to protective effects against the development of new-onset symptomatic HFpEF, left ventricular diastolic dysfunction, and hypertrophy in patients with type 2 diabetes mellitus and hypertension (18). This suggests that the effects of MET in HFpEF could be attributed to its role in improving metabolic flexibility and reducing cardiac stress, offering a potentially unique mechanism for its therapeutic benefit in HFpEF patients. Additionally, Halabi et al. found that metformin treatment was correlated with a decrease in mortality among individuals with HFpEF (19).

The cardiovascular protective effects of EMPA and MET in heart failure have been demonstrated in studies, with notable improvements in prognostic outcomes observed in patients with HFpEF. Despite the abundance of evidence supporting the cardiovascular benefits of these drugs in HFpEF patients, the precise mechanisms remain unclear. Given the differences in the molecular targets of EMPA and MET, it is crucial to explore how these drugs affect distinct metabolic and molecular pathways that contribute to the pathophysiology of HFpEF. This study will help clarify these mechanisms and provide insights into their potential complementary roles in HFpEF therapy.

This study utilizes a “two-hit” induced HFpEF mouse model, which closely replicates the key characteristics of human HFpEF. HFpEF in this model is confirmed through functional, histological, and molecular assessments. The study will evaluate and compare the effects of EMPA and MET on cardiac structure and function, with particular focus on myocardial fibrosis, hypertrophy, and diastolic function. This approach aims to provide valuable insights into the distinct pharmacological mechanisms of these two drugs in the context of HFpEF.

## 2 Materials and methods

### 2.1 Animals

For mice, all the experiments were approved in advance by the Institutional Animal Care and Use Committee of Hunan Provincial People's Hospital (First Affiliated Hospital of Hunan Normal University) (Biomedical Research Ethical Approval number: Lunlun Examination Section 2022 No. 98). Eight-week old male C57BL/6 mice (18–22 g) were purchased from Hunan Silaike Jingda Experimental Animal Co. Ltd., Changsha, Hunan, China (animal qualification certification: No.430727221100933173). The mice were maintained in a controlled environment with temperatures ranging from 20°C to 26°C, relative humidity levels between 30% and 70%, and a 12 h light-dark cycle, while having unrestricted access to tap water and a certified pellet diet.

To ensure no selection bias between the experimental and control groups, the mice were randomly assigned to treatment groups prior to the start of the experiment using a computer-generated randomization list. The allocation process was performed by a researcher who was not involved in subsequent data collection and analysis, and blinding was applied to ensure the fairness of the experiment. For treatment, metformin (MET) (HY-B0627, MedChemExpress LLC, China) and empagliflozin (EMPA) (HY-15409, MedChemExpress LLC, China) were administrated by gavage at doses of 200 mg/kg/d (20) and 10 mg/kg/d (21) respectively. The utilization of animals in research adhered to national regulations governing experimental animal use.

### 2.2 “Two-hit”-induced hFpEF mouse model

The mouse HFpEF model established in this study is based on the double-hit hypothesis proposed by Schiattarella GG et al., which combines metabolic stress and mechanical stress to simulate the pathophysiological conditions of HFpEF (22). In this model, the first “hit” is metabolic stress induced by an 8-week regimen of a 60% high-fat diet (HFD; XTHF60, Xietong Shengwu Co., Ltd, China). The second “hit” is mechanical stress, achieved through the administration of 0.5 g/L N[w]-nitro-l-arginine methyl ester (L-NAME; HY-18729A, MedChemExpress LLC, China) in the drinking water of male C57BL/6J mice. Doppler echocardiography was then conducted at the 8-week mark to assess left ventricular diastolic function and left ventricular ejection fraction (LVEF), in order to confirm the successful construction of the HFpEF model. Successful HFpEF was confirmed based on the following criteria (23): (1) a reduced E/A ratio (<1.0), indicating diastolic dysfunction, and (2) an increased E/E’ ratio (>5 or >8), indicating elevated left ventricular filling pressure.

### 2.3 Pathological morphological staining

The harvested hearts were fixed in 4% buffered formaldehyde for 24 h, embedded in paraffin, and sectioned into 5 µm thick slices. Hematoxylin-eosin staining (H&E staining) and Masson`s trichrome staining were conducted. Heart sections were examined under a microscope to identify collagen deposition, which appeared as blue staining indicative of fibrosis. The quantification of collagen in the heart sections was performed using Leica Microsystems and Image J software. Collagen deposition was quantified by measuring the collagen-positive area within the left ventricular myocardium, and the percentage of collagen fiber area was calculated using the formula: Proportion of collagen fiber area (%) = (Collagen pixel area/Total pixel area of visual field) × 100%. Five different image fields were selected from each section to ensure representative sampling, and the average value was used for statistical analysis.

Wheat germ agglutinin (WGA) staining was used to label the cell membranes of cardiomyocytes. The cross-sectional areas of cardiomyocytes in the left ventricle (LV) were visualized using WGA staining at a concentration of 5 µg/ml (Servicebio, China) and quantified by measuring single myocyte cross-sectional areas with ImageJ software (National Institutes of Health, USA). A minimum of five fields per section were analyzed, and individual cardiomyocyte areas were outlined in each image using ImageJ software. The cross-sectional area of each cardiomyocyte was calculated in mm^2^, and the mean value was obtained from the measurements of all analyzed fields for each sample.

### 2.4 Exercise exhaustion test

Three days prior to the commencement of the experiment, the treadmill (ZH-PT/5S, ZHENGHUA BIO. China) was configured to operate on a 0° incline. Upon completion of the 12-week period, the treadmill was adjusted to a 20° incline, with an electric shock intensity of 0.8 mA, and an initial speed of 5 m/min. Following 4 min of running, the warm-up speed was raised to 14 m/min, with subsequent increments of 2 m/min every 2 min until the mice reached exhaustion. Exhaustion was operationally defined as the point at which the mice received electric shocks 20 times within 10 s or when they could no longer maintain the running speed. The total running distance was then recorded as the measure of exercise endurance.

### 2.5 Glucose tolerance (GTT) and insulin resistance test (ITT)

One week before the experiment, the mice were handled and pre-stimulated by gently stroking their tails. Mice underwent an overnight fast prior to the determination of baseline blood glucose levels (mg/dl) using 10 μl of tail vein blood in a glucose meter (Sinocare, China). Intraperitoneal injection of glucose (2 mg dextrose/g body weight) in sterile PBS was administered, and blood glucose levels were monitored at various time points (0, 15, 30, 60, 120 min) post-injection.

Insulin tolerance testing was performed using the same glucometer after a 6 h fast. Following establishment of baseline glucose values, mice received an intraperitoneal injection of insulin (0.75 U/kg) to assess insulin sensitivity. Clearance of plasma glucose was subsequently monitored at the indicated times post-injection. GTT and ITT tests were performed 1 week apart.

### 2.6 Conventional echocardiography and Doppler imaging

Echocardiography was conducted utilizing an animal ultrasound imaging system (VINNO 6, Beijing, China). Anesthesia was administered with isoflurane, with an initial induction at 5% followed by maintenance at 2%. Left ventricular ejection fraction and other parameters of systolic function were derived from short axis M-mode scans at the mid-ventricular level identified by the presence of papillary muscles in anesthetized mice. Diastolic function measurements were obtained using pulsed-wave mode and tissue Doppler under apical 4-chamber view. Parameters were collected, some of which include heart rate (HR), left ventricular end-diastolic diameter (LVIDd), left ventricular end-systolic diameter (LVIDs), end-diastolic interventricular septal wall thickness (IVSd), left ventricular end-diastolic posterior wall (LVPWd), left ventricular fractional shortening (LVFS), left ventricular ejection fraction (LVEF), peak Doppler blood inflow velocity across the mitral valve during early diastole (E), peak Doppler blood inflow velocity across the mitral valve during late diastole (A), and peak tissue Doppler of myocardial relaxation velocity at the mitral valve annulus during early diastole (E’). Following the completion of the procedures, all mice successfully emerged from anesthesia without any complications. Each parameter was measured a minimum of three times, and the resulting averages are provided.

### 2.7 Plasma NT-proBNP levels were measured by ELISA

After blood collection, the blood in the centrifuge tube was mixed and centrifuged at 2,000 g for 10 min at 4 ℃. 2103;. The upper plasma was taken to measure the level of NT-proBNP in serum according to the instructions of the kit (JL11641, Jianglai biology, China).

### 2.8 Real-Time quantitative polymerase chain reaction analysis

According to the manufacturer's instructions, total RNA was extracted from tissues using Trizol reagent (Invitrogen, Shanghai, China). First-strand cDNA was synthesized using the RevertAid First Strand cDNA Synthesis Kit (Thermo Fisher Scientific, Shanghai, China) with oligo(dT)18 primers. Real-time polymerase chain reaction was conducted in an ABI ViiA 7 Real-Time PCR System using the FastStart Universal SYBR Green Master (Roche, Shanghai, China). The amplification protocol included 1 cycle at 95°C for 10 min, followed by 40 cycles of 95°C for 30 s, 60°C for 1 min, and 72°C for 1 min. A melting curve analysis was performed post-amplification to ensure the specificity of the amplified product. The primers used in this study are listed in Table 1.

**Table 1:** RT-PCR primer sequence.

Gene	Primer sequence (5’-3’)	
IL1B	Sense	GAAATGCCACCTTTTGACAGTG
	Antisense	TGGATGCTCTCATCAGGACAG
NFKB1	Sense	ATGGCAGACGATGATCCCTAC
	Antisense	CGGAATCGAAATCCCCTCTGTT
MAP2K1	Sense	GAGTGCAACTCCCCGTACATC
	Antisense	TTCTCCCGAAGATAGGTCAGG
PRKACA	Sense	GGTGACAGACTTCGGTTTTGC
	Antisense	CACAGCCTTGTTGTAGCCTTT
HSP90aa1	Sense	AATTGCCCAGTTAATGTCCTTGA
	Antisense	CGTCCGATGAATTGGAGATGAG
*β*-actin	Sense	GGCTGTATTCCCCTCCATCG
	Antisense	CCAGTTGGTAACAATGCCATGT

### 2.9 Bioinformatics analysis

To identify potential targets for EMPA, MET, and HFpEF, bioinformatics analysis was performed using several online databases. Specifically, we used Swiss Target Prediction (http://www.swisstargetprediction.ch/), Super-PRED (http://prediction.charite.de/), Genecards (https://www.genecards.org/), Inheritance in Man (OMIM) (https://omim.org/), and Comparative Toxicogenomics Databases (http://ctdbase.org/) to filter and identify relevant targets. These tools help predict drug-target interactions and provide comprehensive gene information, which is essential for understanding the molecular mechanisms involved in HFpEF. The targets were then selected based on their biological relevance to the pathophysiology of HFpEF, particularly focusing on genes related to inflammation, fibrosis, and cardiac remodeling.

### 2.10 Western blot analysis

For protein analysis in cardiac tissues, samples were homogenized and centrifuged at 14,000 rpm for 15 min at 4°C. Protein concentrations in the supernatant were determined using the Pierce BCA Protein Assay Kit (Thermo Fisher Scientific). Following SDS-PAGE separation, 50 µg of protein was transferred onto a PVDF membrane. The membrane was blocked with 5% skim milk at room temperature for 1 h and then washed with TBST. Rabbit monoclonal antibodies against HSP90, TGFβ, and β-actin (dilution 1:1000) were added and incubated overnight at 4°C. After another washing step, the appropriate secondary antibodies were applied, and the ECL kit was used for visualization. The grey values of the protein bands were analyzed using the Gel Imaging System, and relative protein expression levels were normalized to β-actin as an internal control.

### 2.11 Statistical analysis

SPSS 26.0 software was used for analysis, GraphPad 9.0 was used to plot, and the data were expressed as mean ± standard deviation. Data were first tested for normality using Shapiro–Wilk Test, while homogeneity of variance was tested using Leven's Test. For normality, Shapiro–Wilk Test was applied for sample sizes less than 50, and Kolmogorov–Smirnov Test was applied for larger sample sizes. The Levene's Test was performed to assess homogeneity of variance. The *t* test was used to compare the data with normal distribution and homogeneity of variance between two samples, and one-way analysis of variance was used to compare the data between multiple samples. In the case of significant differences in variances, Welch's ANOVA was used for comparison of multiple groups. If the data did not follow a normal distribution or had uneven variances, the nonparametric Mann–Whitney U test or the Kruskal–Wallis test combined with Dunn's test was used to test the significance of the differences. Multiple comparisons were controlled using the Bonferroni correction for parametric tests and the Dunn's test for non-parametric tests. Missing data were handled by using mean imputation for small amounts of missing values, and outliers were identified using the Grubbs’ test and excluded from the analysis. Differences were considered statistically significant if *P* < 0.05.

To minimize potential bias, all data analyses were performed without knowledge of group assignments. The analysts did not have access to group information during data processing and statistical analysis, ensuring the fairness and reliability of the analysis process.

## 3 Results

### 3.1 HFpEF mouse model was created by feeding high-fat diet and L-NAME

**Figure 1:**
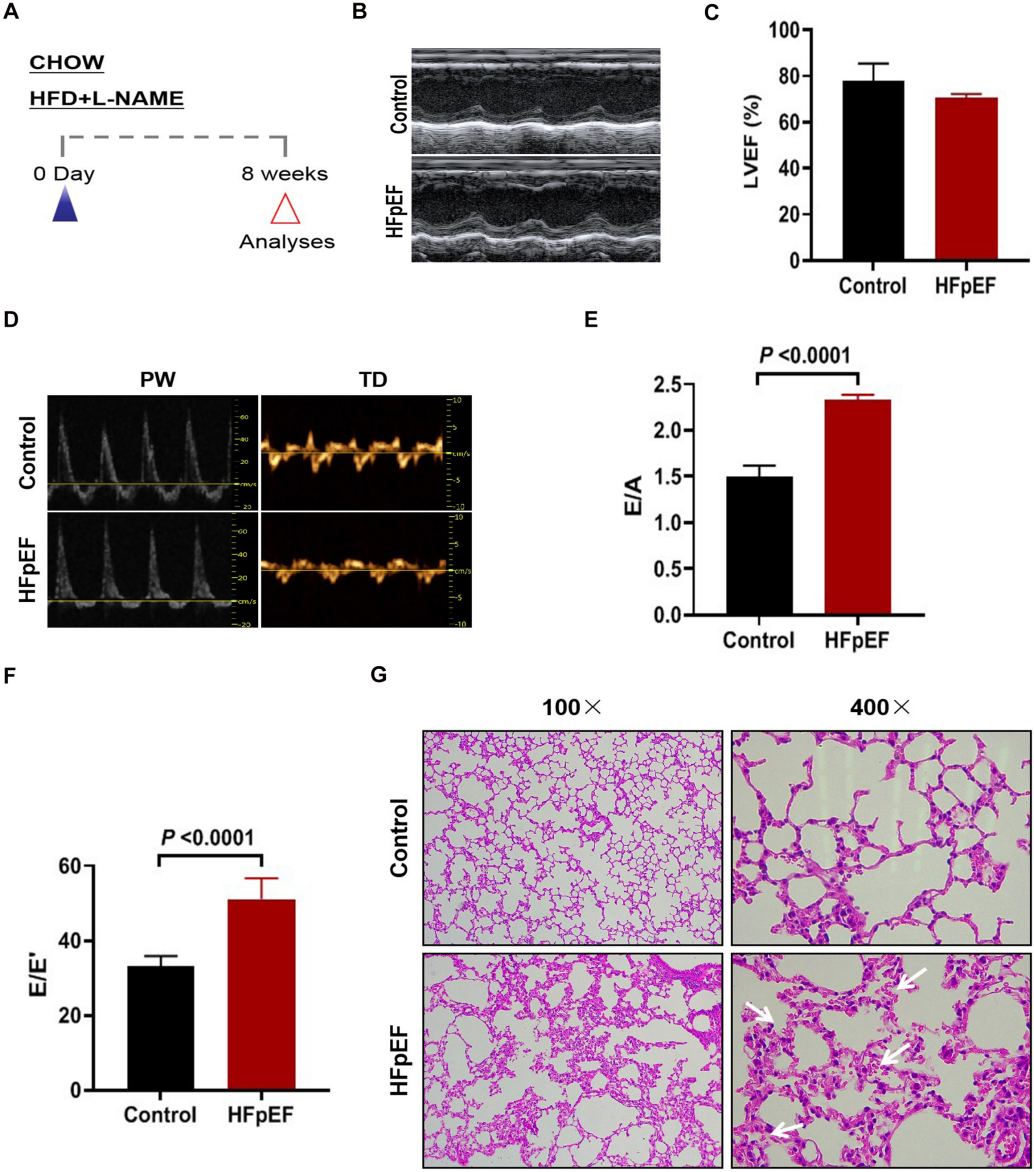
Construction of hFpEF mouse model. **(A)** Experimental design. C57BL/6J mice were maintained on different dietary regimens (filled triangle) and followed up to the eighth week (empty red triangles). **(B)** The representative LV M-mode echocardiographic tracings. **(C)** Percent LV ejection fraction (LVEF%). **(E)** The representative pulsed-wave Doppler (top) and tissue Doppler (bottom) tracings. **(D)** Ratio between mitral E wave and A wave(E/A). **(F)** Ratio between mitral E wave and E’ wave (E/E’). **(G)** Lung tissue was stained with H&E, where blue arrows indicate massive red blood cell infiltration (Control group: *n* = 5; Experimental group: *n* = 4).

A “two-hit” approach was employed to establish an animal model of HFpEF, in which C57BL/6J mice were subjected to a HFD and L-NAME administration (Figure 1A). Echocardiographic assessment demonstrated sustained preservation of left ventricular ejection fraction (LVEF) in HFpEF mice while compared to control mice (Figures 1B,C). However, different levels of diastolic dysfunction were detected between the control mice and the HFpEF mice, showing elevated the ratio of E/A and E/E’ in HFpEF mice as indicated by noninvasive Doppler (Figures 1E,F). Concurrently, an assessment was conducted on the pathological morphology of lung tissue in two groups of mice. The findings indicated that HFpEF mice exhibited notable pathological alterations, including thickened alveolar walls, congested capillaries, and reduced alveolar cavity, suggesting pulmonary congestion (Figure 1G). These results indicated that the HFpEF mouse model was successfully constructed.

### 3.2 The effect of EMPA and MET on body weight and blood pressure in HFpEF mice

**Figure 2:**
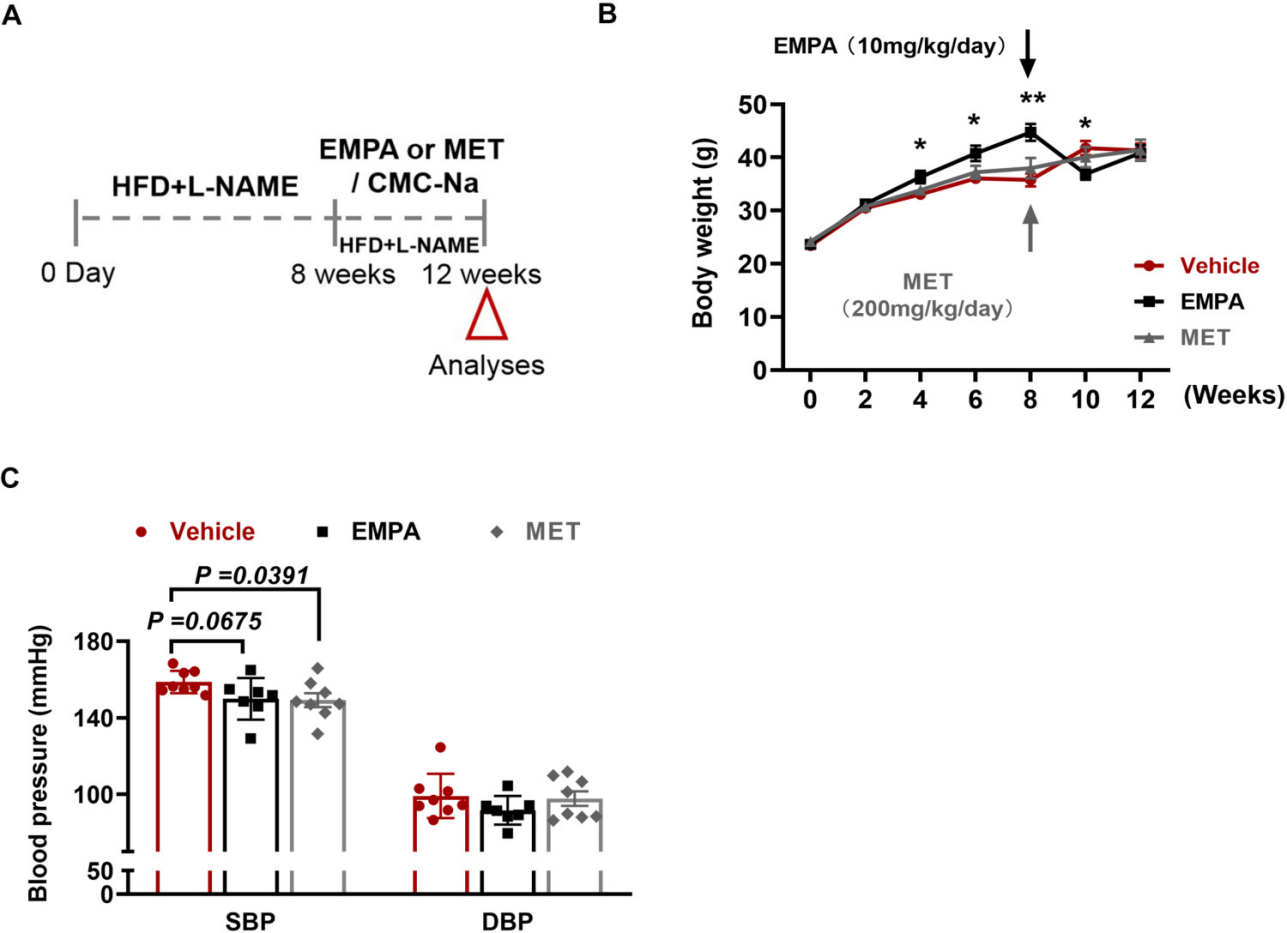
Role of EMPA and MET in body weight and blood pressure in hFpEF mice. **(A)** Experimental design. C57BL/6J mice were maintained on dietary regimens to the eighth week, and then EMPA and EMT were given intragastric administration respectively for continuous 4 weeks, followed up to the twelveth week (empty red triangles). The mice only given a same volume of 0.5% CMC-Na as vehicle group. **(B)** Body weight changes in HFpEF mice following EMPA (10 mg/kg/day) or MET (200 mg/kg/day) treatment vs. Vehicle. **(C)** Blood pressure changes (SBP and DBP) in HFpEF mice following EMPA or MET treatment vs. Vehicle (Vehicle group: *n* = 7; EMPA group: *n* = 7; EMT group: *n* = 8).

The impact of EMPA and MET on cardiac structure and function in mice with HFpEF was assessed through oral administration for a duration of 4 weeks (Figure 2A). Firstly, we evaluated the effects of EMPA and MET on body weight and blood pressure in HFpEF mice. Following EMPA administration, a significant decrease in body weight was observed at weeks 8 and 10, whereas MET intervention did not yield any notable effect on body weight changes (Figure 2B). MET administration resulted in an average reduction of 9.5 mmHg in systolic blood pressure(SBP), but presented no impact on the diastolic blood pressure (DBP) in HFpEF mice. While EMPA exhibited a tendency to lower systolic blood pressure in HFpEF mice, the difference was not deemed statistically significant (Figure 2C).

### 3.3 The effect of EMPA and MET on glucose metabolism in HFpEF mice

**Figure 3:**
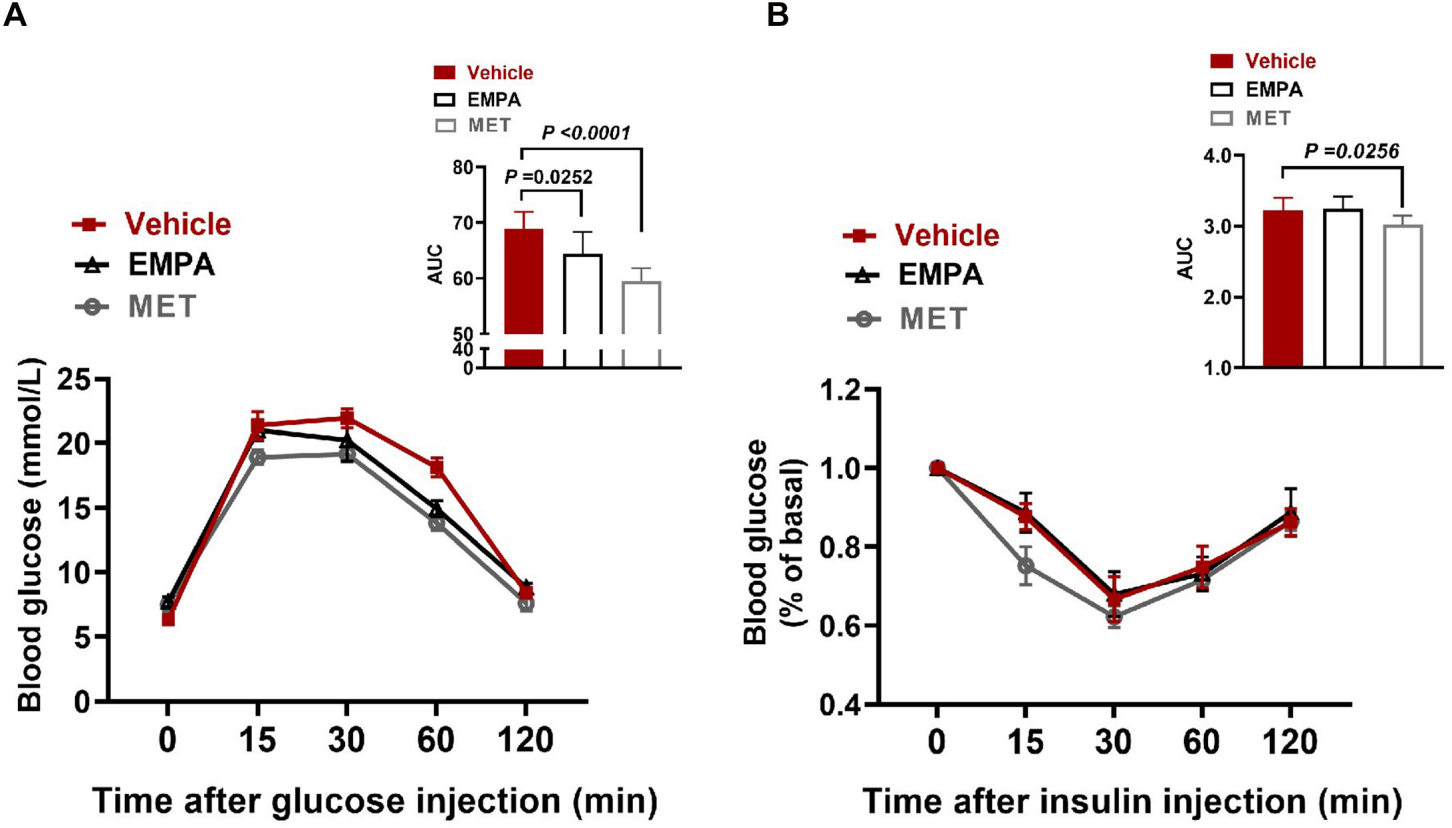
Role of EMPA and MET in glucose metabolism in hFpEF mice. **(A)** Blood glucose changes and Area under curve (AUC) in glucose tolerance test (GTT) between EMPA group and Vehicle group. **(B)** Blood glucose changes and AUC in Insulin tolerance testing (ITT) between EMPA group and Vehicle group. Vehicle group: *n* = 7; EMPA group: *n* = 7; MET group: *n* = 8.

Subsequently, we evaluated the effects of EMPA and MET on glucose metabolism, and the results showed that both EMPA and MET improved glucose tolerance in HFpEF mice (Figure 3A). In addition, MET also improved insulin sensitivity in HFpEF mice (*P* = 0.0256) (Figure 3B), while EMPA treatment did not improve insulin sensitivity *in vivo*, suggesting that MET has a stronger effect on improving glucose metabolism.

### 3.4 The effect of EMPA and MET on cardiac function and exercise tolerance in HFpEF mice

**Figure 4:**
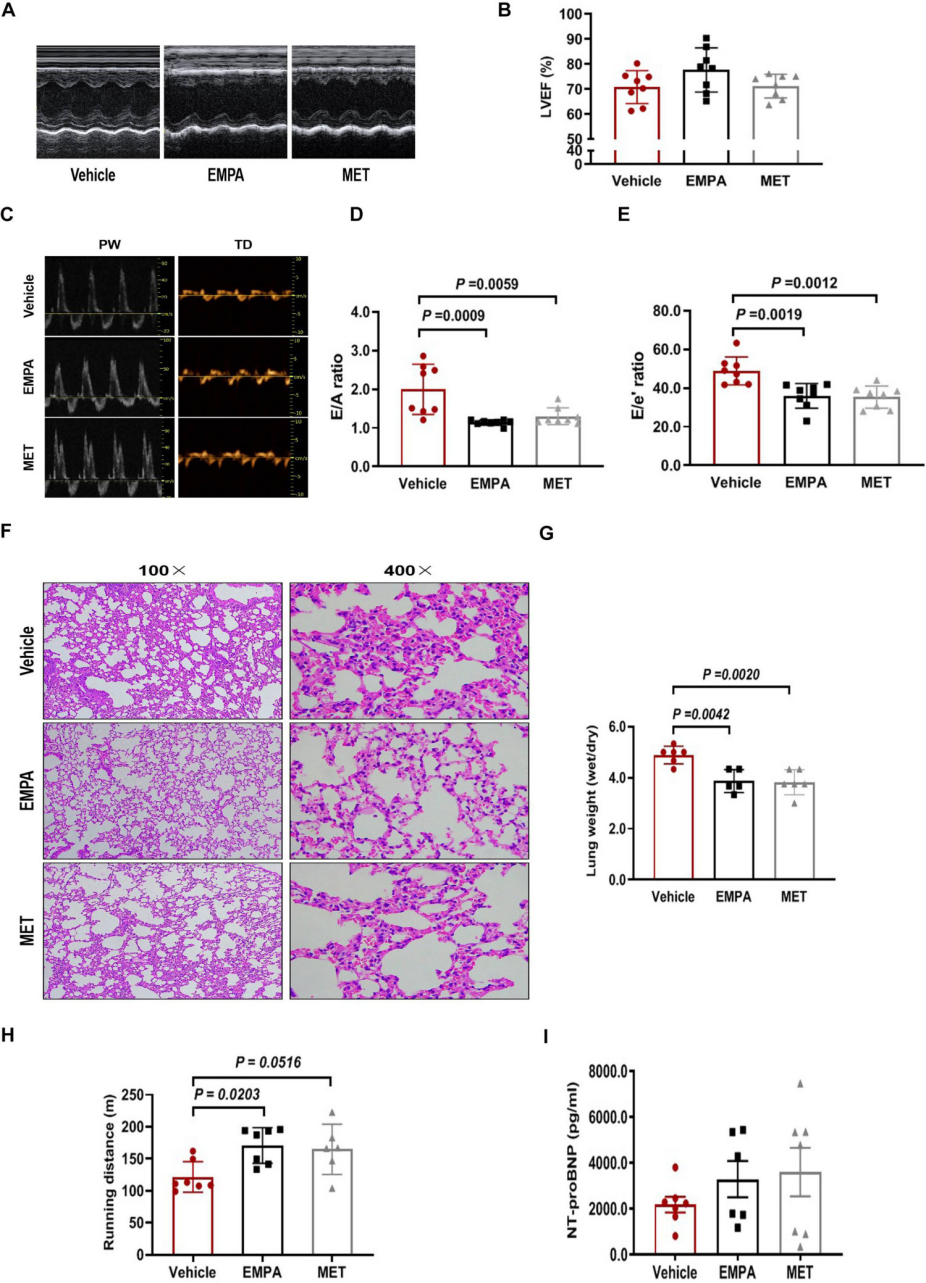
Role of EMPA and MET in cardiac function and exercise tolerance in hFpEF mice. **(A)** The representative LV M-mode echocardiographic tracings. **(B)** Percent LV ejection fraction (LVEF%). **(C)** The representative pulsed-wave Doppler (top) and tissue Doppler (bottom) tracings. **(D)** Ratio of E/A. **(E)** Ratio of E/E’. **(F)** Lung tissue was stained with H&E. **(G)** The whole lung was weighed as wet after using gauze to remove residual blood and fluid from the lung surface. Subsequently, the tissues were subjected to a drying process in an oven at 80°C for 48 h until a constant weight was achieved, after which the dry weight was measured to determine the W/D ratio. **(H)** Exercise exhaustion test in each group. **(I)** The levels of plasma NT-proBNP. Vehicle group: *n* = 7; EMPA group: *n* = 7; MET group: *n* = 8.

Echocardiographic assessment demonstrated EMPA and MET have no significant effect on LVEF in HFpEF mice (Figures 4A,B). However, we observed a remarkable improvement on cardiac diastolic function both in EMPA-treated and MET-treated HFpEF mice, showing significant reduction on the E/A and E/E’ ratios (Figures 4C,E). Additionally, HE staining showed that the alveolar wall congestion was significantly ameliorated in the MET group and EMPA group, and the Dry-Wet weight (D/W) ratio of the whole lung was significantly reduced (Figures 4F,G). These data indicate that both EMPA and EMT could effectively enhance cardiac diastolic function and reduce pulmonary congestion in HFpEF mice, but no significant difference among their pharmacological effects is observed *in vivo* in this two-hit HFpEF mouse model.

We then examined the effects of EMPA and MET on aerobic/endurance exercise in HFpEF mice. Compared to the vehicle group, EMPA could increase the exercise exhaustion distance by 49.2 m averagely in HFpEF mice. Although MET also showed a trend to improve exercise endurance in HFpEF mice, the difference was not statistically significant (Figure 4H). These results suggest that while EMPA significantly improves endurance exercise in HFpEF mice compared to the vehicle, it does not show a statistically significant improvement over MET.

The level of N-segment B-type natriuretic peptide precursor (NT-proBNP) in ventricular secretion plasma is positively correlated with left ventricular diastolic filling pressure **(**24, 25). However, we found no significant difference in plasma NT-proBNP levels among vehicle, EMPA and MET groups (Figure 4I).

### 3.5 The effect of EMPA and MET on cardiac structure in HFpEF mice

**Figure 5:**
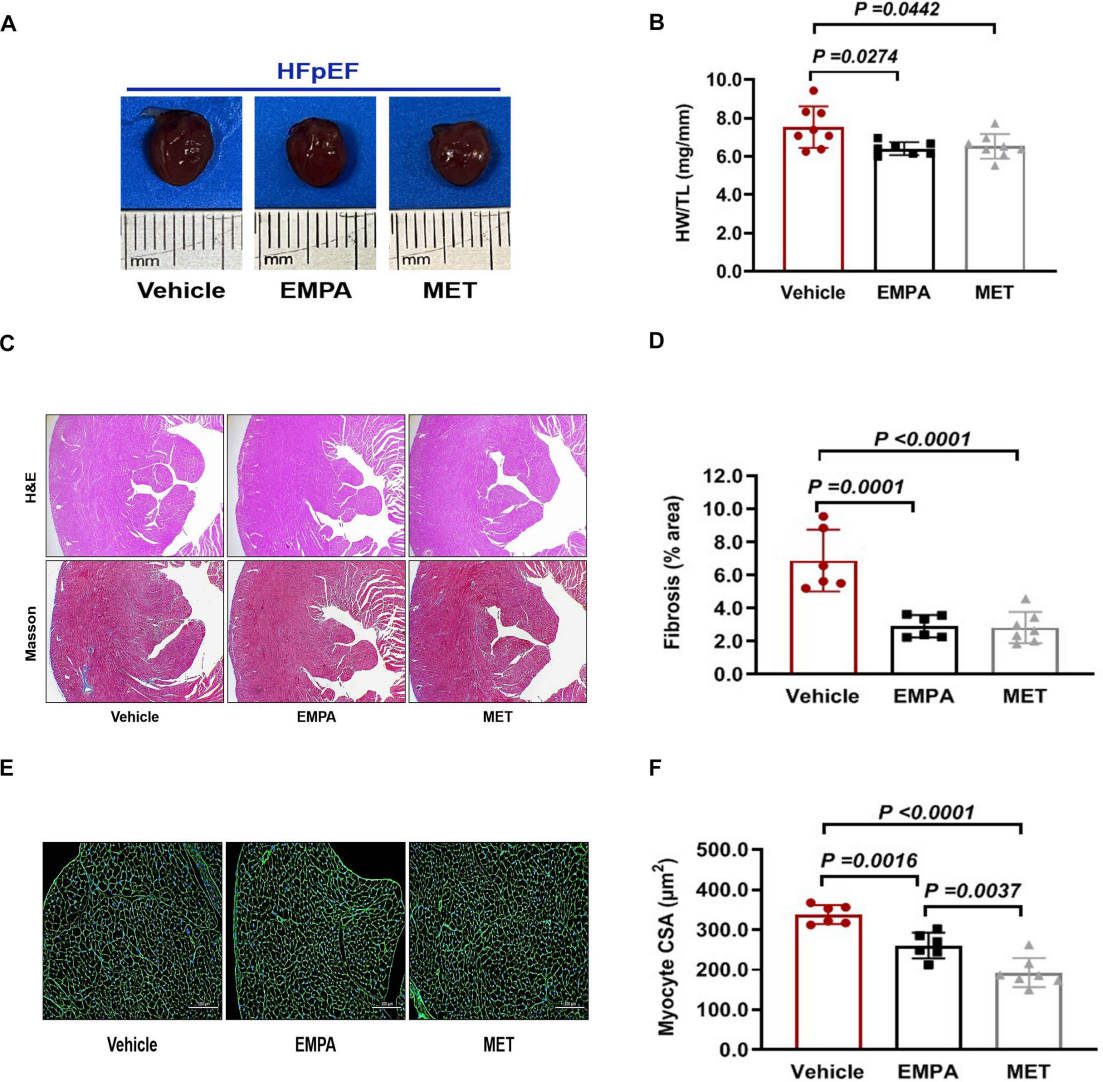
Role of EMPA and MET in cardiac structure in hFpEF mice. **(A)** The representative gross view of heart from each group. **(B)** The heart weight (HW) and tibial length (TL) were quantified, and the ratio of HW to TL was subsequently computed. **(C)** The representative images of H&E and Masson staining of Heart. **(D)** The proportion of collagen fiber area was calculated. **(E)** The representative images of WGA staining of Heart. **(F)** The cross sectional area (CSA) of cardiomyocyte analyzed by Image J Pro software. Vehicle group: *n* = 7; EMPA group: *n* = 7; MET group: *n* = 8.

We further evaluated the effects of EMPA and MET on cardiac stucture in HFpEF mice. We found a reduction in the heart weight/tibial length (HW/TL) ratio in HFpEF mice after EMPA or EMT treatment. However, there was no statistically significant difference observed between the EMPA and MET groups (Figures 5A,B). Both EMPA and MET were found to significantly inhibition of myocardial fibrosis (Figures 5C,D). Furthermore, wheat germ agglutinin (WGA) staining revealed improvements in the cross-sectional area of cardiomyocytes in MET-treated or EMPA-treated HFpEF mice, but a more pronounced effect was observed in the MET group compared to the EMPA group (Figures 5E,F). Based on the above results, both EMPA and MET could improve cardiac remodeling including fibrosis and hypertrophy in HFpEF mice, and MET was generally more effective than EMPA.

### 3.6 Simplified mechanistic analysis of EMPA and MET in HFpEF: target identification and functional insights

**Figure 6:**
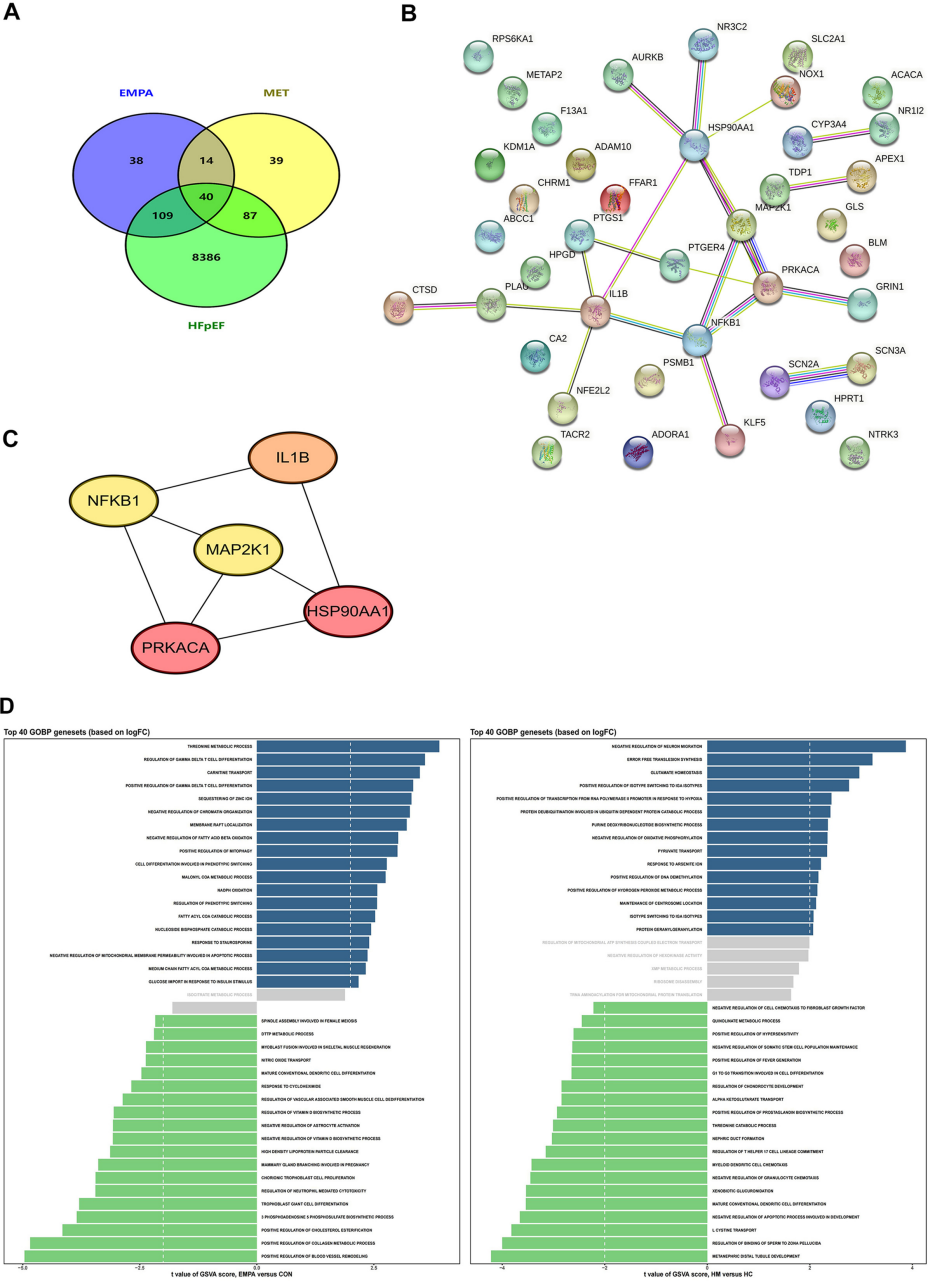
Pharmacological mechanism of EMPA and MET in the treatment of heart failure explored through network pharmacology analysis. **(A)** Venn diagram of the intersection targets between EMPA, MET, and heart failure with preserved ejection fraction. **(B)** Protein-protein interaction network of the intersection targets analyzed using the STRING database. **(C)** Five key core genes of EMPA and MET acting on heart failure, screened using the CytoHubba plugin and MCC algorithm in Cytoscape software. **(D)** GO analysis of the intersection targets performed using R packages (clusterProfiler, enrichplot, and ggplot2).

To comprehensively elucidate the therapeutic mechanisms of EMPA and MET in HFpEF, we filtered the targets for EMPA, MET and HFpEF using the Swiss Target Prediction, Super-PRED, Genecards, Inheritance in Man (OMIM), and Comparative Toxicogenomics Databases. The core genes were selected based on their biological relevance to the pathophysiology of HFpEF, with a focus on genes implicated in critical processes such as inflammation, fibrosis, and cardiac remodeling. Genes were chosen if they were known to interact with EMPA and MET and had documented expression in the heart, particularly those that regulate myocardial fibrosis and hypertrophy, which are hallmark features of HFpEF.Through Venn diagram analysis, we discovered 40 overlapping targets (Figure 6A), which may be effective targets for EMPA and MET in the treatment of HFpEF. To further refine the selection, we focused on genes that had high confidence protein-protein interactions (PPI) in the STRING database, with a threshold of >0.7, ensuring that the interactions are reliable and directly relevant to HFpEF. Next, to understand the interactions between these targets, we used the STRING database for protein-protein interaction (PPI) network analysis. The interaction network of potential targets in HFpEF comprised 21 nodes and 21 edges at a high confidence of >0.7 (Figure 6B). By using Cytoscape software (Version: 3.10.2) in conjunction with MCC algorithms of the CytoHubba plugin, we identified the top 5 hub genes within the network (IL1B, NFKB1, MAP2K1, PRKACA, and HSP90AA1), suggesting that these genes are potential targets for EMPA and MET in heart failure therapy (Figure 6C).

To further analyze the distinct biological effects of EMPA and MET, we performed a Gene Ontology Biological Process (GOBP) term analysis on the top 40 gene sets based on logFC. The results showed that EMPA treatment significantly upregulated the positive regulation of collagen metabolic processes, which refers to the processes involved in the synthesis, degradation, and remodeling of collagen fibers within the extracellular matrix (Figure 6D). This indicates that EMPA has a positive impact on collagen synthesis. As a crucial component of the extracellular matrix, collagen plays a key role in maintaining cardiac structure and function. Therefore, the upregulation of collagen metabolic processes induced by EMPA treatment may enhance its efficacy in HFpEF therapy by promoting cardiac tissue remodeling and repair.

### 3.7 Validation experiments for gene and protein expression analysis

**Figure 7:**
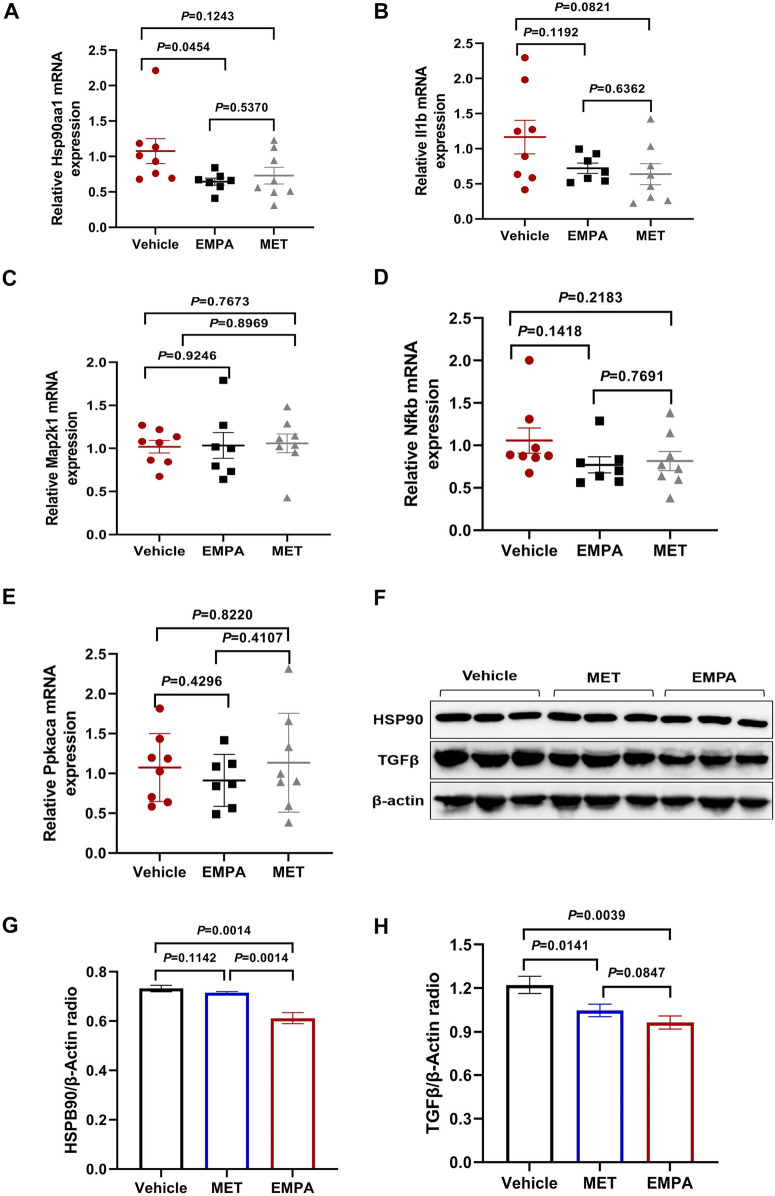
Expression analysis of Key genes and proteins in heart failure models treated with EMPA and MET. **(A–E)** Relative mRNA expression levels of Hsp90aa1 **(A)**, Il1b **(B)**, Map2k1 **(C)**, Nfkb1 **(D)**, and Prkaca **(E)** in heart tissue from vehicle, EMPA, and MET treated groups. Data are presented as the mean ± SEM. **(F)** Representative Western blot images showing the protein expression levels of HSP90, TGFβ, and β-actin in heart tissue from vehicle, EMPA, and MET treated groups. **(G–H)** Quantification of Western blot results. Protein levels of HSP90 **(G)** and TGFβ **(H)** were normalized to β-actin. Data are presented as the mean ± SEM (Vehicle group: *n* = 7; EMPA group: *n* = 7; MET group: *n* = 8).

To further investigate the mechanisms of action of EMPA and MET in the treatment of HFpEF, we analyzed the mRNA expression of key genes (*Hsp90aa1*, *Il1b*, *Map2k1*, *Nfkb*, *Prkaca*). The results showed that EMPA treatment significantly reduced *Hsp90aa1* mRNA expression compared to the control group, while the mRNA expression levels of *Il1b*, *Map2k1*, *Nfkb*, and *Prkaca* did not show significant differences (Figures 7A–E). Complementary to these findings, Western Blot analysis revealed that EMPA significantly reduced HSP90 expression (*P* = 0.0014), whereas MET had no significant impact on HSP90 expression (*P* = 0.1142) (Figures 7F,G). Furthermore, both EMPA and MET significantly reduced TGFβ expression (*P* = 0.0141 and *P* = 0.0039, respectively), but the difference between the two was not significant (*P* = 0.0847) (Figures 7F,H). These results suggest that EMPA may exert its therapeutic effects in HFpEF by specifically downregulating the expression of HSP90 and TGFβ, while MET primarily acts by downregulating TGFβ. In conclusion, these findings provide important insights for further research into the specific mechanisms of EMPA and MET in the treatment of HFpEF.

## 4 Discussion

HFpEF, a prevalent form of heart failure, is characterized by a multifaceted pathophysiological mechanism, encompassing various disease mechanisms. Recent research indicates that HFpEF is linked to numerous risk factors including advanced age, sex, hypertension, pulmonary congestion, metabolic syndrome, obesity, type 2 diabetes, hyperlipidemia, and renal insufficiency. These risk factors or comorbidities interact in a complex manner within the pathophysiological framework of HFpEF (26–28). Consequently, the diagnosis of HFpEF poses significant challenges. In recent years, two scoring models, HFA-PEFF and H2FPEF (29), have been created to enhance the precision and consistency of diagnosing HFpEF. These models have undergone validation in contemporary clinical investigations and comprehensively encapsulate the clinical characteristics of HFpEF patients (30). The mouse model utilized in this research study displayed manifestations consistent with HFpEF, such as pulmonary congestion, diminished exercise tolerance, and a high burden of comorbidities including hypertension, obesity, and prediabetes. The study demonstrated that HFpEF models were developed through the combined application of a high-quality diet and L-NAME. The HFA-PEFF score was determined to be 6 points, while the H2FPEF score was 4 points according to these scoring models (26). Additionally, the experimental findings presented in this paper confirmed the high level of concordance between this modeling approach and the clinical phenotype of HFpEF.

Our research findings indicate that while MET did not lead to a reduction in body weight in mice with HFpEF, this discrepancy may stem from the different molecular targets and mechanisms of action between MET and EMPA. Clinical studies have shown that MET, particularly in obese individuals with type 2 diabetes, can result in weight loss (31), yet in our study, the lack of weight reduction may reflect the specific interaction of MET with metabolic pathways in HFpEF mice. Additionally, our observations regarding the weight-reducing effects of EMPA in HFpEF mice align with previous research by Xu et al. (32), and this effect could be due to EMPA's ability to induce browning of white adipose tissue, which may promote thermogenesis and energy expenditure, offering a plausible explanation for the observed weight reduction in HFpEF mice. The observed decrease in SBP in HFpEF mice following treatment with MET aligns with findings from Borg et al.'s investigation involving type 2 diabetic patients (33). MET has been shown to lower postprandial SBP, potentially through mechanisms involving glucagon-like peptide-1 and delayed gastric emptying. Results from the EMPEROR-Reduced trial indicated no significant interaction between EMPA and SBP (34), further supporting the conclusions drawn in the present study. The findings of this study demonstrate that both MET and EMPA have the potential to enhance glucose metabolism in mice with HFpEF, a conclusion supported by existing clinical research (35). Furthermore, the stronger impact of MET on glucose metabolism compared to EMPA in our study may be attributed to MET's direct effects on insulin sensitivity, a mechanism that may be more relevant in the HFpEF model, which shares metabolic abnormalities with type 2 diabetes. Additionally, the study suggests that both MET and EMPA may be beneficial in mitigating risk factors and complications associated with HFpEF, including hypertension, obesity, and impaired glucose tolerance.

This study demonstrated that MET has the potential to decrease the E/A and E/È ratios, ultimately enhancing cardiac diastolic function in mice with HFpEF. Slater et al. further elucidated that MET may reduce the passive stiffness of actin, suggesting a potential mechanism for the improvement in diastolic function (36). Additionally, the study revealed that EMPA also exhibited positive effects on diastolic function in HFpEF mice, potentially through an increase in the phosphorylation level of myofilament regulatory proteins and a decrease in myofilament passive stiffness, ultimately leading to an improvement in cardiac diastolic function (37). Although both EMPA and MET improved diastolic function, the mechanisms of action may differ, with EMPA potentially enhancing myofilament regulation while MET may exert its effects through structural changes to the actin filaments. When comparing the pharmacological effects of the two drugs, EMPA demonstrated a more pronounced effect on improving exercise endurance in HFpEF mice, with a significant increase in exercise exhaustion distance.This suggests that EMPA may have additional benefits in improving exercise tolerance compared to MET, which showed a trend but no statistically significant difference. Interestingly, the study revealed that neither MET nor EMPA had a significant impact on plasma NT-proBNP levels. Specifically, MET did not demonstrate any effect on plasma NT-proBNP levels, and the cardioprotective mechanism of MET in HFpEF mice could not be attributed to its influence on plasma NT-proBNP (38). Furthermore, the EMPEROR-Preserved trial, which assessed the impact of EMPA on plasma NT-proBNP levels in HFpEF patients, indicated that the adjusted mean difference in plasma NT-proBNP levels between EMPA and placebo was only 7% (39). The mean duration of EMPA administration exceeded 2 years, while the drug intervention period in this study lasted 4 weeks. It is postulated that EMPA may demonstrate efficacy in reducing plasma NT-proBNP levels in HFpEF mice following an extended duration of drug intervention.

While we acknowledge the statistical limitations of this study, particularly the lack of formal power analysis and corrections for multiple hypothesis testing, which may have affected the statistical significance and increased the risk of false positives, our findings still provide valuable insights into the potential of MET and EMPA in HFpEF treatment. We plan to incorporate power analysis and appropriate corrections for multiple hypothesis testing in future studies to further enhance the robustness of our results.

This study, through various bioinformatics and experimental methods, revealed the potential mechanisms of EMPA and MET in the treatment of HFpEF. The differences observed in the mechanisms of EMPA and MET—such as EMPA's effect on collagen metabolism and MET's influence on TGFβ signaling—may explain the differing therapeutic outcomes between the two drugs, which warrants further investigation into their complementary roles in HFpEF treatment. HSP90 is a molecular chaperone that plays a pivotal role in protein folding, stability, and function (40). HSP90 has been shown to stabilize proteins involved in the activation of pro-fibrotic and inflammatory signaling, contributing to cardiac fibrosis and dysfunction (41, 42). In this study, we found that EMPA downregulated the expression of HSP90, which may contribute to its therapeutic effects by reducing fibrosis and improving cardiac remodeling. TGFβ, a key regulator of fibrosis, is involved in the activation of fibroblasts and the deposition of extracellular matrix components (43, 44). The downregulation of TGFβ observed in both EMPA and MET treatments may help mitigate the fibrotic remodeling commonly seen in HFpEF. These findings highlight the roles of HSP90 and TGFβ in the progression of HFpEF and suggest that targeting these pathways may provide therapeutic benefits in treating the disease.

However, while the study reports changes in HSP90 and TGFβ expression, causality has not been established. We acknowledge this limitation and emphasize that future research, such as using gene knockout or overexpression models, will be necessary to confirm the causal relationship between the expression changes of these molecules and the therapeutic effects observed in HFpEF.

In conclusion, this study established a clinically relevant HFpEF mouse model and evaluated MET and EMPA's effects on cardiac structure and function. Both drugs inhibited fibrosis and hypertrophy, improved diastolic function, and reduced pulmonary congestion. Mechanistically, EMPA upregulated collagen metabolism and downregulated HSP90 and TGFβ, aiding cardiac remodeling and stress reduction. MET primarily downregulated TGFβ, reducing fibrosis. These findings underscore the potential of both MET and EMPA in HFpEF treatment, with EMPA showing additional benefits in improving exercise endurance and collagen metabolism. These results provide a basis for further research and therapeutic development.

## Data Availability

The data analyzed in this study is subject to the following licenses/restrictions: The data is available in fully anonymized form for research purposes upon reasonable request, pending the approval of the study's steering committee. Requests to access these datasets should be directed to rock_kinhool@hotmail.com.
